# Targeting the Mitochondrial Pyruvate Carrier for Neuroprotection

**DOI:** 10.3390/brainsci9090238

**Published:** 2019-09-18

**Authors:** Bor Luen Tang

**Affiliations:** 1 Department of Biochemistry, Yong Loo Lin School of Medicine, National University Health System, Singapore 117596, Singapore; bchtbl@nus.edu.sg; 2 NUS Graduate School for Integrative Sciences and Engineering, National University of Singapore, Singapore 119077, Singapore

**Keywords:** excitotoxicity, mitochondrial pyruvate carrier (MPC), neuroprotection, neurotoxicity, Warburg effect

## Abstract

The mitochondrial pyruvate carriers mediate pyruvate import into the mitochondria, which is key to the sustenance of the tricarboxylic cycle and oxidative phosphorylation. However, inhibition of mitochondria pyruvate carrier-mediated pyruvate transport was recently shown to be beneficial in experimental models of neurotoxicity pertaining to the context of Parkinson’s disease, and is also protective against excitotoxic neuronal death. These findings attested to the metabolic adaptability of neurons resulting from MPC inhibition, a phenomenon that has also been shown in other tissue types. In this short review, I discuss the mechanism and potential feasibility of mitochondrial pyruvate carrier inhibition as a neuroprotective strategy in neuronal injury and neurodegenerative diseases.

## 1. Introduction

Regulatory nodes of energy metabolism have emerged as potential therapeutic targets in multiple human diseases such as cancer, aging and neurodegeneration [1,2,3,4,5,6,7]. The 3-Carbon (C) monocarboxylate pyruvate is a key intermediate in energy metabolism [8]. During aerobic respiration, pyruvate generated by cytosolic glycolysis is shuttled into the mitochondria matrix, decarboxylated into the 2-C acetyl-CoA, and fed into the tricarboxylic acid (TCA) cycle. Pyruvate could alternatively be carboxylated by pyruvate carboxylase in an anaplerotic mode to form oxaloacetate (OAA). The above mitochondrial metabolic modes of pyruvate are dependent on pyruvate import from the cytosol by mitochondrial pyruvate carrier (MPC) [9,10]. Postmitotic brain neurons are energetically intensive cell types [11], and maintenance of the neuronal membrane polarization resting potential under normal functional conditions is very much dependent on efficient ATP generation via oxidative phosphorylation. In other words, a constant supply of pyruvate generated from glucose [12] to the mitochondria is pivotal for neuronal function and survival. In times of heightened activities and injury, lactate provided by the astrocyte-neuron lactate shuttle plays an important role in ensuring pyruvate availability [13]. In fact, a good number of studies have shown that exogenous pyruvate is neuroprotective in hypoglycemic episodes [14], ischemic, epileptic or neurotoxic injuries [15,16,17,18,19], as well as neurodegenerative conditions [20,21]. From another perspective, dysfunctions and abnormalities in mitochondrial energetics underlie many neurological disorders and neurodegenerative diseases [22,23,24], and contributed to neuronal demise during injury [25]. In this regard, improving mitochondrial energetics dysfunction would be beneficial to disease outcome.

In view of the above, it may, therefore, be somewhat non-intuitive but interesting that the inhibition of pyruvate transport by MPC was recently shown to be beneficial in experimental models of neurotoxicity, particularly in the context of Parkinson’s disease (PD) models [26], and is also protective against excitotoxic neuronal death [27]. These findings, and the possibility of MPC inhibition as a neuroprotective strategy in neuronal injury and neurodegenerative diseases, shall be the focus of the discussion below.

## 2. The Mitochondria Pyruvate Carrier

The mechanism underlying mitochondrial cytosolic pyruvate transport into the mitochondrial matrix has been of great interest. However, this is better understood only recently with the identification of the mitochondria pyruvate carriers (MPC) [10,28], which are non-canonical transporters unrelated to the classically recognized carboxylate transporters encoded by the *SLC25A* gene family [9,29]. Cytosolic pyruvate cannot access the mitochondrial matrix by diffusion across the inner mitochondrial membrane (IMM). Early work by Halestrap and colleagues have shown that pyruvate uptake by isolated mitochondria exhibited saturation kinetics and identified specific inhibitors for the process, such as UK-5099 (2-Cyano-3-(1-phenyl-1H-indol-3-yl)-2-propenoic acid) [30], all of which attested to the notion that mitochondrial pyruvate transport occurs via specific carriers [31]. A putative MPC candidate subsequently identified in yeast [32] was, however, later shown to be a NAD^+^ transporter [33]. 

The true MPC paralogues were eventually identified in yeast, *Drosophila* and mouse in 2012 by the laboratories of Rutter [34] and Martinou [35]. Both groups were studying two conserved paralogues with unknown function, Brain protein 44 (BRP44) and BRP44-like (BRP44L) (subsequently human MPC2 and MPC1, respectively). The compelling cellular, biochemical and functional evidences accompanying these works included the severe impairment of mitochondria pyruvate import in yeast Mpc1 mutants, and that expression of mammalian MPC1 and MPC2 in the bacteria *Lactococcus lactis* conferring pyruvate uptake activity characteristic of eukaryotic UK-5099-sensitive mitochondrial pyruvate import [35]. The discovery of MPCs has been extensively reviewed, and interested readers are hereby referred to these excellent accounts [9,28,36].

*S. cerevisiae* has three MPC paralogues, while mouse has two. Placental mammals have an additional paralogue MPC1-like (MPC1L), which is expressed almost exclusively in the testis, particularly in postmeiotic spermatids and sperm cells [37]. MPC has also been described in plants, and the *Arabidopsis* MPC orthologue NRGA1 is a negative regulator of guard cell abscisic acid signaling by affecting cellular pyruvate content [38,39]. Genetic deletion of all three yeast paralogues is tolerated in rich medium, but even the *mpc1*Δ and *mpc2*Δ mutants exhibit measurable growth impairment in non-fermentable carbon sources or with amino acid depletion [35]. Moreover, cellular changes and adaptations in *mpc1*Δ mutants tend to increase oxidative damage of the mitochondria and restrict cell survival during yeast chronological aging [40]. Genetic ablation of either MPC1 [41] or MPC2 [42] in mouse resulted in embryonic lethality. Point mutations in human MPC1 [34,43] are known to result in impairment of pyruvate oxidation with affected individuals presented with developmental abnormality, neurological problems and metabolic deficits. 

Cross-species complementation of *S. cerevisiae mpc1*Δ mutant indicate that Mpc1 function is evolutionarily conserved but has non-overlapping roles with Mpc2. MPC1 and MPC2 could homo- and hetero-oligomerize and form large ~150 kDa complexes. Human MPC2 has been shown to mediate efficient pyruvate transport into proteoliposomes alone, and its expression in Mpc-deficient yeast stimulated growth and increased oxygen consumption [44]. However, the functional unit for MPC’s pyruvate carrier function is most likely a MPC1-MPC2 heterodimer [34,45,46]. For yeast, while Mpc1 expression does not change with different carbon sources, the other two paralogues are specifically expressed under fermentative (Mpc2) or respiratory (Mpc3) conditions [47,48]. The Mpc3-containing carrier complex has higher pyruvate transport activity compared to that containing Mpc2, and alternative Mpc2/3 expression likely serves as an adaptive response to nutrient availability [48].

## 3. Metabolic Insights Arising from the Manipulation of MPC Levels and Activity

The identification of the MPC paralogues has led quickly to some experimental insights on its systemic role in mammalian energy metabolism. One prominent role for MPC is in glucose-stimulated insulin secretion. Pharmacological inhibition or expression silencing of MPC in pancreatic β-cells [49] blocked the glucose-stimulated increase in insulin secretion, while UK-5099 administration caused impaired glucose tolerance [49]. Glucose intolerance is likewise observed for a MPC2 hypomorphic mouse line harboring an N-terminally truncated protein, a phenotype that could be attributed to an impairment in glucose-stimulated pancreatic insulin released [42]. In this connection, a particularly important finding is that the anti-diabetic drug family of thiazolidinediones (TZDs), known previously as peroxisome proliferator-activated receptor gamma (PPARγ) agonists [50,51], turned out to be MPC inhibitors at clinically relevant concentrations [45,52]. MPC1 and MPC2 were identified as mitochondrial proteins that could be chemically cross-linked to TZD in a manner that could be blocked by UK-5099, thus the term “mitochondrial target of TZDs” (mTOT) [52,53]. In fact, a TZD analog MSDC-0602, with very low affinity for binding and activation of PPAR*γ*, could nonetheless exert significant pharmacological effects on insulin sensitivity, and improve metabolic derangements in mice with liver-specific PPARγ knockout [54]. MPC inhibition by TZD also underlies its stimulation of glucose uptake in myotubes and myocytes, and UK-5099 mimicked TZD’s activation of AMP-activated protein kinase (AMPK) [45]. These findings attested to the importance of mitochondrial pyruvate uptake as a key regulatory node in acute glucose sensing and uptake. 

On the other hand, defective mitochondrial pyruvate uptake is also associated with aberrant metabolism in diseased cells and tissues. For example, cancer cells have altered energy metabolism, often termed the Warburg effect [55,56], which is associated with a tendency for cancerous cells to generate lactate from pyruvate and have reduced aerobic oxidation even under normoxic and aerobic conditions. The Warburg effect has been associated with an elevation in pyruvate kinase isoform M2 [57] and a decrease in pyruvate production, an upregulation of lactate dehydrogenase (LDH) [58], or an inhibition of pyruvate dehydrogenase (PDH) by the induction of pyruvate dehydrogenase kinase 1 (PDK1) under hypoxic conditions common to solid tumors [59]. However, the Warburg effect could also be due to a loss of MPC1 and consequential impairment of mitochondrial pyruvate transport in multiple cancer types [60,61,62,63,64,65,66,67]. MPC1 expression and activity appears to limit malignancy [60,68], suppress the expressions of stemness markers [60,64,69,70], and influences epithelial-mesenchymal transition [66,71]. On the other hand, MPC inhibition has been shown to have an antitumor effect, in blocking lactate uptake and sensitizing tumor xenografts to radiotherapy [72].

Given the apparently critical importance of mitochondrial pyruvate transport, would there be sufficient room for maneuver in engaging MPCs as drug targets? Would MPC inhibition detrimentally diminish ATP production or critically paralyze the TCA cycle’s energetic and anaplerotic [73] roles? Studies with MPC silencing or inhibition with cells in culture have in fact indicated a surprising degree of cellular metabolic flexibility and adaptation associated with acute loss of MPC activity. While loss of MPC resulted in a significant reduction in both glucose and pyruvate oxidation, cell growth, oxygen consumption, and the TCA cycle functionality in general were apparently not drastically affected. Yang and colleagues showed using SFxL glioma cells [74] that pyruvate import suppresses mitochondrial glutamate dehydrogenase (GDH) and glutamine-dependent acetyl-CoA formation [75]. MPC inhibition by UK-5099 activated GDH, thus rerouting glutamine metabolism to generate both oxaloacetate and acetyl-CoA to maintain TCA cycle function, as well as for lipid synthesis [75]. Vacanti and colleagues also showed that for C2C12 myoblasts with MPC knockdown, TCA flux was maintained by enhanced glutaminolysis through the malic enzyme and pyruvate dehydrogenase (PDH), as well as through fatty acid and branched-chain amino acid oxidation [76]. 

More recent studies have also demonstrated systemic effects that are non-detrimental with tissue type specific *MPC* knockouts. Gray and colleagues showed that liver-specific knockout of MPC1 markedly decreased pyruvate-driven gluconeogenesis and induced adaptive utilization of glutamine. Constitutive hepatic MPC1 deletion attenuated the development of hyperglycemia induced by a high-fat diet. Even acute MPC1 deletion in hepatocytes (in *MPC* floxed (*MPC1^fl/fl^)* littermates treated with AAV-Cre driven by a hepatocyte-specific promoter) after diet-induced obesity decreased hyperglycemia and improved glucose tolerance [77]. McCommis and colleagues found that mice with a liver-specific knockout of MPC2 had impaired conversion of labeled pyruvate to TCA cycle intermediates and glucose [78], which impairs gluconeogenesis and apparently protects the animals from hyperglycemia. Blood glucose levels were in fact maintained by pyruvate-alanine cycling, and glucose production by MPC2-deficient hepatocytes could thus be effectively reduced by inhibition of pyruvate-alanine transamination [78]. On the other hand, Sharma and colleagues showed that muscle-specific MPC deletion in mouse diverted pyruvate mitochondrial import into circulating lactate and increased muscle fatty acid oxidation and decreased adiposity [79]. In fact, muscle glucose uptake and whole-body insulin sensitivity were also increased. Importantly, these studies show that targeted inhibition of MPC may be beneficial to pathological conditions of obesity and hyperglycemia associated with type 2 diabetes mellitus (T2DM). They would also suggest that the antidiabetic effects of TZDs may act, at least partially, through MPC inhibition.

## 4. MPC Inhibition and Neuroprotection

Considering the effect of MPC loss or inhibition on metabolic changes at the cellular and organismal level, it is conceivable that MPC inhibition may confer benefits in certain diseased conditions. However, for postmitotic brain neurons that are critically dependent on glucose or lactate fueled high intensity energy metabolism and ATP production for their survival and functionality, pyruvate is known to be neuroprotective in different neuropathological contexts. Whether acute MPC inhibition could even be tolerated by brain neurons is doubtful. It is therefore somewhat surprising that MPC inhibition has been shown to confer protective benefits in two somewhat different aspects of neuroprotection, namely dopaminergic neuronal death in PD models and excitotoxic neuronal death due to glutamate. We now turn to these cases.

### 4.1. MPC Inhibition and Protection against Neurotoxic Compounds and Factors

Ghosh and colleagues have examined the effect of the MPC-specific TZD, MSDC-0160, in dopaminergic neuron protection using several different PD models [26]. The authors found that MSDC-0160 protected tyrosine hydroxylase (TH)-expressing differentiated Lund human mesencephalic (LUHMES) cells, TH-positive mouse primary mesencephalic neurons in culture, as well as GFP-labelled dopaminergic neurons in *C. elegans*, against the neurotoxicity of 1-methyl-4-phenylpyridinium (MPP^+^). MSDC-0160 also rescued dopaminergic neurons in *C. elegans* expressing the human PD-associated A53T mutant of *α*-synuclein from degeneration. In mouse, systemically administered MSDC-0160 could effectively enter brain tissues. In a PD mouse model in which animal brains were lesioned with the MPP^+^ precursor 1-methyl-4-phenyl-1,2,3,6-tetrahydropyridine (MPTP), MSDC-0160 treatment, either pre- or post-MPTP administration elicited an array of beneficial effects. Locomotor behavioural function, nigral dopaminergic neuron survival, and striatal dopamine levels were improved while neuroinflammation was reduced. In another mouse model in which heterozygocity of Engrailed1 (*En1*^+/−^) produces a progressive nigrostriatal degeneration phenotype [80], MSDC-0160 administration from either 3 or 8 weeks of age significantly preserved motor function, TH-positive neuron viability, striatal content of dopamine and its metabolite, as well as reduced neuroinflammation. Thus, it appears that MPC inhibition protects against classical neurotoxins like MPTP and its active metabolite MPP^+^, as well as neurotoxicity incurred by mutant α-synuclein.

How exactly does MSDC-0160 exert its neuroprotective effect as an MPC inhibitor? Ghosh and colleagues hypothesized that neuroprotection may involve the mechanistic target of rapamycin (mTOR) pathway, which functions downstream of MPC activity [81]. mTOR inhibition has indeed been associated with benefits in aging and neurodegeneration [82], particularly through modulation of autophagy [83]. In this regard, Ghosh and colleagues have also shown that MSDC-0160′s beneficial effects were dependent on a non-acute, negative modulation of the mTOR pathway as demonstrated by reduced phosphorylation of mTOR and its substrate p70S6 kinase, which likely resulted from the more acute perturbative action on mitochondrial metabolism. Furthermore, MSDC-0160 induced mitochondrial metabolic changes could likely reduce astrogliosis and microgliosis by reducing NF-κB based inflammatory signaling in glial cells in a manner that also involves mTOR inhibition, with the latter having been shown to downregulate brain neuroinflammation in other contexts [84,85,86]. In summary, MPC inhibition could be neuroprotective via a mTOR signaling modulation in both a neuron cell-autonomous manner through metabolic changes and adaptation, as well as a reduction of neuroinflammation mediated by glial cells.

### 4.2. MPC Inhibition and Protection against Glutamate Excitotoxicity

In another key report, Divakaruni and colleagues investigated the effect of MPC inhibition in excitotoxic neuronal death [27]. The authors found that unlike the respiratory chain inhibitor antimycin A (a cytochrome c reductase inhibitor) which causes death, rat cortical neurons maintained in culture with the MPC inhibitor UK-5099 remain viable for 3 days or more despite having their pyruvate oxidizing capacity reduced by more than half. Interestingly, the neurons appeared to rather readily oxidize other substrates, such as leucine and β-hydroxybutyrate, when supplemented in customized media during UK-5099 treatment. These non-glucose substrates could reverse alterations in glycolytic rate and total ATP production with MPC inhibition, and stable isotope tracings showed that these non-glucose substrates contributed significantly to the acetyl-CoA pool and TCA cycle intermediates. In rich media, incorporation of glucose-derived C into the TCA cycle intermediates was significant reduced byUK-5099 treatment, but basal metabolic rate is maintained and there was no substantial elevation of cellular lactate levels. Interestingly, however, oxidation of leucine and β-hydroxybutyrate were also not substantially increased during MPC inhibition in rich medium, indicating that pyruvate transport inhibited neurons have adapted metabolically by oxidising another substrate. 

A profiling of metabolites and TCA cycle intermediates showed, among expected changes in citrate and alanine, UK5099 treatment reduced total glutamate levels by half, whereas there was a twofold increase in aspartate. This suggests an increased in oxidation of glutamate (or glutamine), which could occur via aspartate aminotransferase or oxidative deamination by glutamate dehydrogenase [87,88], and has been demonstrated in other cell/tissue types with MPC inhibition [75,77,89]. In neurons, this is not known to occur to any significant degree as neuronal cell glutamate is usually associated with the neurotransmitter pool at the synaptic compartments. Isotope tracing with labelled glutamine showed that UK-5099 treatment indeed increased glutamine uptake and C fluxes of glutamine-derived Acetyl-CoA to TCA cycle intermediates. The authors also hypothesized that the preferred oxidation of glutamate, but not other substrates, could reflect a cellular adaptation to compensate for loss in pyruvate-based anaplerosis. This notion is supported by the observation that the labelled third C of the [3-^13^C1] glucose tracer found in TCA cycle intermediates were significantly diminished by UK-5099. The reciprocal regulation of pyruvate and glutamate oxidation could also be discerned to some degree in UK-5099 treated organotypic hippocampal slice cultures with mixed neural cell types. 

Perhaps the most intriguing finding of Divakaruni and colleagues is that this glutamate oxidation promoted by MPC inhibition could ultimately be neuroprotective. Glutamate is an excitatory neurotransmitter, and its uncontrolled release during massive glutamatergic neuron depolarization, resulting from acute oxygen-glucose depletion accompanying ischemic or traumatic injury, mediates excitotoxic neuronal death. Use of neuronal glutamate for oxidation may conceivably decrease the glutamate released upon depolarization, and could thus limit excitotoxic neuronal injury. Indeed, neurons treated with UK-5099 released less glutamate extracellularly upon depolarization induced by veratridine (which prevents sodium channel closure), ouabain (which inhibits Na^+^/K^+^-ATPase) and an N-methyl aspartate (NMDA) pulse. Furthermore, cortical neurons treated with UK-5099 were protected from excitotoxic cell death by glutamate added in culture medium in a non-additive manner to the protective effects of the excitotoxicity-mediating NMDA receptor antagonist MK801. Post-synaptic currents in neurons treated with UK-5099 also produced a smaller current density peak in excitatory postsynaptic current elicited by a hypertonic sucrose shock. Together, these findings therefore suggest that enhancing glutamate oxidation by MPC inhibition could indeed attenuate the glutamate pool released upon depolarization. 

## 5. Caveats and Unanswered Questions

**Figure 1:**
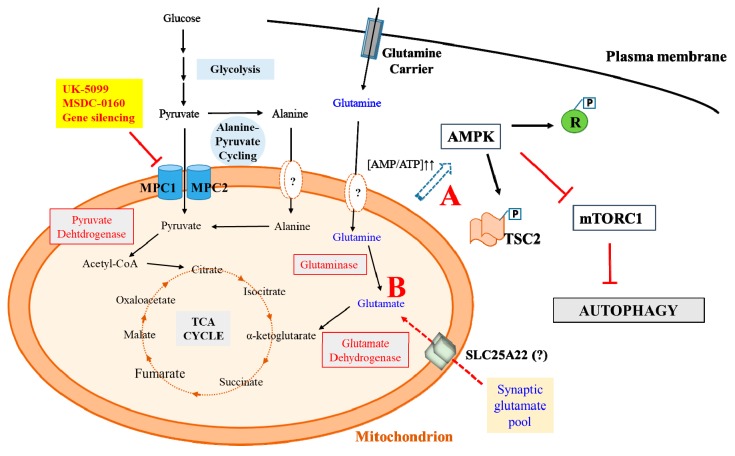
A schematic diagram summarizing the pathways and possible mechanisms underlying mitochondrial pyruvate carrier (MPC) inhibition-mediated neuroprotection that has recently came to light. Metabolic adaptations in a neuronal cell that underlie neuroprotection could result in an Inhibition of mechanistic target of rapamycin complex 1 (mTORC1) via AMP-dependent protein kinase (AMPK) activation due to increased AMP/ATP ratio (**A**), which promotes neuroprotective autophagy. AMPK could inhibit mTORC1 by phosphorylating Raptor (R) or Tuberous sclerosis complex 2 (TSC2). Metabolic adaptations could also promote a switch to oxidizing alternative substrates like glutamate (**B**), which may reduce the synaptic glutamate pool, thus attenuating excitoxicity. Glutamate feeding into tricarboxylic acid (TCA) cycle (conversion of glutamate to *α*-ketoglutarate by glutamate dehydrogenase sustains the anaplerotic needs of the cell. Reduced mitochondrial pyruvate import and feeding into the TCA cycle may conceivably also be compensated by enhanced alanine-pyruvate cycling.

The above findings indicating that MPC inhibition is neuroprotective gave rise to many intriguing questions. Although MSDC-0160-mediated neuroprotection could be largely attributed to MPC inhibition, it is yet unclear exactly how the protective benefit arises. Ghosh and colleagues have made observations that are suggestive of inhibition of mTOR signaling. However, whether this accounts for the neuroprotective effect was not confirmed. Furthermore, how mTOR signaling, particularly through the mTORC1 complex [90], could be affected by MPC inhibition is yet unclear. One possibility is the elevation of AMP/ATP ratio activated AMP-dependent kinase (AMPK) [91] (see Figure 1). AMPK could suppress mTORC1 signaling through the phosphorylation of Raptor [92], thus excluding it from the functional mTORC1 complex, or the phosphorylation of the tumor suppressor Tuberous sclerosis complex 2 (TSC2) [93], a key negative regulator of mTORC1 in conjunction with TSC1 [94]. Exerting neuroprotection via AMPK activation [95,96] is also a major mechanism underlying the neuroprotective effect of another first-line diabetic drug, the dimethylbiguanide metformin [97,98]. Given the complexity of metabolic adaptation that ensues upon MPC inhibition, whether neuroprotection could be largely explained by inhibition of mTORC1 through AMPK activation needs further experimental verification.

Pertaining to the findings of Divakaruni and colleagues, the details of metabolic adaptation resulting from inhibition of mitochondrial pyruvate transport leading to an enhanced glutamate oxidation are also unclear. There are several questions that need to be addressed. Firstly, despite the inhibition of mitochondrial import, could mitochondrial pyruvate levels be nonetheless sustained by other pathways? In the classically known Cahill cycle (or glucose-alanine cycle) [99] between muscle and liver, muscles degrade amino acids for energy needs with alanine transaminase (ALT), which converts glutamate and pyruvate into α-ketoglutarate and alanine. ALT is present in both cytosol and the mitochondria and could potentially sustain the level of mitochondrial pyruvate using alanine. However, its expression is limited to tissues like muscle and liver, and whether there are any significant ALT activities in neurons is unclear. Another possible way to sustain mitochondrial pyruvate is the conversion of malate to pyruvate by malate dehydrogenase, with malate generated from glutamine or glutamate’s conversion to *α*-ketoglutarate by glutamate dehydrogenase (GDH), thus feeding into the TCA cycle (Figure 1). A switch from fueling the TCA cycle with glutamine rather than glucose is indeed a feature of cancer cells [100]. 

The data of Divakaruni and colleagues appear to suggest that the synaptic glutamate pool of glutamatergic neurons may plausibly equilibrate with that of the mitochondria, thus increasing mitochondrial glutamate. How this might occur is not particularly clear. The mitochondrial glutamate carrier 1 (SLC25A22) [101] is mainly astrocytic, and whether it is upregulated in neurons as a result of MPC inhibition to mediate glutamate import into the mitochondrial would be a point that deserves further investigation. In γ-aminobutyric acid (GABA)ergic neurons, glutamine is actively taken up as a precursor for GABA synthesis. Glutamine can be converted to glutamate by glutaminase, and in theory therefore, the same mechanism of alternative substrate oxidation could sustain MPC-inhibited GABAergic neurons as well. How different neuronal types adapt to an inhibition of mitochondrial pyruvate transport would be of significant interest in the immediate future. 

## 6. Is MPC Inhibition a Viable Strategy for Neuroprotection?

Given the critical role of MPCs in mammalian development and the problems of patients harboring MPC mutations, MPC inhibition might be perceived as being overly detrimental for cellular and organismal energetics and survival to be of benefit. However, as the findings above in liver, muscle and neurons have demonstrated, genetic ablation or pharmacological inhibition of MPC activities could be contextually beneficial in diseased conditions. This is very much down to the previously underappreciated metabolic adaptability exhibited under the condition of mitochondrial pyruvate transport inhibition. As far as neuropathological conditions are concerned, a metabolic shift to the usage of glutamine/glutamate that would reduce glutamate release and excitotoxicity, as well as the possibility of mTOR pathway inhibition that could lead to elevated autophagy and suppression of neuroinflammation, would constitute neuroprotective benefits. The reprogramming of mitochondrial metabolism resulting from MPC inhibition could therefore provide context-dependent benefits under neuronal disease conditions, which could potentially also be extended to diseases in other organs or tissues.

There are also indications that MPC inhibition could be beneficial in other neuropathological conditions. TZDs have been shown to be beneficial for a range of neurodegenerative diseases, including Alzheimer’s disease (AD) [102,103,104,105], PD [106,107,108,109,110], Amyotrophic lateral sclerosis [111,112], and Huntington’s disease [113,114,115]. AD’s pathological connections with T2DM has in particular led to it being termed as Type 3 diabetes mellitus [116], and emerging data on the underlying mechanisms in cognitive decline or dementia due to brain insulin resistance [117,118] would point towards TZDs being potentially disease-modifying or symptom-alleviating in AD [119]. Recent meta-analyses have also indicated that pioglitazone use reduced stroke events in T2DM patients [120], and is also associated with a reduced incidence of secondary stroke [121,122]. Furthermore, pioglitazone administration in rat chronic TBI model improved working memory [123]. Although the neuroprotective and beneficial effects of the TZDs in neuropathology are usually attributed to PPARγ activation, in retrospection their inhibition of MPCs may have been at least partially responsible.

What then, are the clinical prospects of MPC inhibition in neuroprotection? TZDs which are standard diabetic drugs with ample clinical testing already done, have been and should be further investigated in trials [124]. The aerobic glycolysis-inhibiting cancer drug lonidamine has also been shown to be a potent MPC inhibitor [125], has low toxicity [126,127] and has been used in multiple clinical trials in cancer [126,128]. Given that lonidamine inhibits MPC with a Ki of 2.5 µM and an IC50 of ~20 µM [125] and that plasma concentrations exceeding its IC50 could be easily achieved in patients with little toxicity [126], its used for neurological conditions appears feasible. Long-term use of stronger inhibitors such as UK-5099 (IC50, 50 nM) may not be feasible but its acute application may be tolerated. This would need to be determined in preliminary trials.

## 7. Conclusions

As an epilogue, therefore, findings in neuroprotection resulting from MPC inhibition are intriguing. Although there is much more to be learned before the underlying mechanisms are fully explained and the strategy could be implemented effectively and safely, the translational potential is fairly obvious.

## References

[1] Yin F., Sancheti H., Patil I., Cadenas E. (2016). Energy metabolism and inflammation in brain aging and Alzheimer’s disease. Free Radic. Biol. Med..

[2] Camandola S., Mattson M.P. (2017). Brain metabolism in health, aging, and neurodegeneration. EMBO J..

[3] Bose S., Ramesh V., Locasale J.W. (2019). Acetate metabolism in physiology, cancer, and beyond. Trends Cell Biol..

[4] Icard P., Fournel L., Wu Z., Alifano M., Lincet H. (2019). Interconnection between metabolism and cell cycle in cancer. Trends Biochem. Sci..

[5] Papadopoli D., Boulay K., Kazak L., Pollak M., Mallette F., Topisirovic I., Hulea L. (2019). mTOR as a central regulator of lifespan and aging. F1000Research.

[6] Sasaki Y. (2019). Metabolic aspects of neuronal degeneration: From a NAD+ point of view. Neurosci. Res..

[7] Quansah E., Peelaerts W., Langston J.W., Simon D.K., Colca J., Brundin P. (2018). Targeting energy metabolism via the mitochondrial pyruvate carrier as a novel approach to attenuate neurodegeneration. Mol. Neurodegener..

[8] Gray L.R., Tompkins S.C., Taylor E.B. (2014). Regulation of pyruvate metabolism and human disease. Cell. Mol. Life Sci..

[9] Palmieri F., Monné M. (2016). Discoveries, metabolic roles and diseases of mitochondrial carriers: A review. Biochim. Biophys. Acta.

[10] Bender T., Martinou J.C. (2016). The mitochondrial pyruvate carrier in health and disease: To carry or not to carry?. Biochim. Biophys. Acta.

[11] Dienel G.A. (2017). The metabolic trinity, glucose-glycogen-lactate, links astrocytes and neurons in brain energetics, signaling, memory, and gene expression. Neurosci. Lett..

[12] Mergenthaler P., Lindauer U., Dienel G.A., Meisel A. (2013). Sugar for the brain: The role of glucose in physiological and pathological brain function. Trends Neurosci..

[13] Mason S. (2017). Lactate shuttles in neuroenergetics-homeostasis, allostasis and beyond. Front. Neurosci..

[14] Choi B.Y., Kim J.H., Kim H.J., Yoo J.H., Song H.K., Sohn M., Won S.J., Suh S.W. (2013). Pyruvate administration reduces recurrent/moderate hypoglycemia-induced cortical neuron death in diabetic rats. PLoS ONE.

[15] Izumi Y., Zorumski C.F. (2010). Neuroprotective effects of pyruvate following NMDA-mediated excitotoxic insults in hippocampal slices. Neurosci. Lett..

[16] Ryou M.G., Liu R., Ren M., Sun J., Mallet R.T., Yang S.H. (2012). Pyruvate protects the brain against ischemia-reperfusion injury by activating the erythropoietin signaling pathway. Stroke.

[17] Moro N., Ghavim S.S., Harris N.G., Hovda D.A., Sutton R.L. (2016). Pyruvate treatment attenuates cerebral metabolic depression and neuronal loss after experimental traumatic brain injury. Brain Res..

[18] Popova I., Malkov A., Ivanov A.I., Samokhina E., Buldakova S., Gubkina O., Osypov A., Muhammadiev R.S., Zilberter T., Molchanov M. (2017). Metabolic correction by pyruvate halts acquired epilepsy in multiple rodent models. Neurobiol. Dis..

[19] Tanaka K.I., Shimoda M., Kawahara M. (2018). Pyruvic acid prevents Cu2+/Zn2+-induced neurotoxicity by suppressing mitochondrial injury. Biochem. Biophys. Res. Commun..

[20] Isopi E., Granzotto A., Corona C., Bomba M., Ciavardelli D., Curcio M., Canzoniero L.M.T., Navarra R., Lattanzio R., Piantelli M. (2015). Pyruvate prevents the development of age-dependent cognitive deficits in a mouse model of Alzheimer’s disease without reducing amyloid and tau pathology. Neurobiol. Dis..

[21] Wang X., Hu X., Yang Y., Takata T., Sakurai T. (2015). Systemic pyruvate administration markedly reduces neuronal death and cognitive impairment in a rat model of Alzheimer’s disease. Exp. Neurol..

[22] Rose S., Niyazov D.M., Rossignol D.A., Goldenthal M., Kahler S.G., Frye R.E. (2018). Clinical and molecular characteristics of mitochondrial dysfunction in Autism Spectrum Disorder. Mol. Diagn. Ther..

[23] Wu Y., Chen M., Jiang J. (2019). Mitochondrial dysfunction in neurodegenerative diseases and drug targets via apoptotic signaling. Mitochondrion.

[24] Cowan K., Anichtchik O., Luo S. (2019). Mitochondrial integrity in neurodegeneration. CNS Neurosci. Ther..

[25] Zhao X.Y., Lu M.H., Yuan D.J., Xu D.E., Yao P.P., Ji W.L., Chen H., Liu W.L., Yan C.X., Xia Y.Y. (2019). Mitochondrial dysfunction in neural injury. Front. Neurosci..

[26] Ghosh A., Tyson T., George S., Hildebrandt E.N., Steiner J.A., Madaj Z., Schulz E., Machiela E., McDonald W.G., Escobar Galvis M.L. (2016). Mitochondrial pyruvate carrier regulates autophagy, inflammation, and neurodegeneration in experimental models of Parkinson’s disease. Sci. Transl. Med..

[27] Divakaruni A.S., Wallace M., Buren C., Martyniuk K., Andreyev A.Y., Li E., Fields J.A., Cordes T., Reynolds I.J., Bloodgood B.L. (2017). Inhibition of the mitochondrial pyruvate carrier protects from excitotoxic neuronal death. J. Cell Biol..

[28] McCommis K.S., Finck B.N. (2015). Mitochondrial pyruvate transport: A historical perspective and future research directions. Biochem. J..

[29] Taylor E.B. (2017). Functional properties of the mitochondrial carrier system. Trends Cell Biol..

[30] Hildyard J.C.W., Ammälä C., Dukes I.D., Thomson S.A., Halestrap A.P. (2005). Identification and characterisation of a new class of highly specific and potent inhibitors of the mitochondrial pyruvate carrier. Biochim. Biophys. Acta.

[31] Thomas A.P., Halestrap A.P. (1981). Identification of the protein responsible for pyruvate transport into rat liver and heart mitochondria by specific labelling with [3H] *N*-phenylmaleimide. Biochem. J..

[32] Hildyard J.C.W., Halestrap A.P. (2003). Identification of the mitochondrial pyruvate carrier in Saccharomyces cerevisiae. Biochem. J..

[33] Todisco S., Agrimi G., Castegna A., Palmieri F. (2006). Identification of the mitochondrial NAD+ transporter in Saccharomyces cerevisiae. J. Biol. Chem..

[34] Bricker D.K., Taylor E.B., Schell J.C., Orsak T., Boutron A., Chen Y.C., Cox J.E., Cardon C.M., Van Vranken J.G., Dephoure N. (2012). A mitochondrial pyruvate carrier required for pyruvate uptake in yeast, Drosophila, and humans. Science.

[35] Herzig S., Raemy E., Montessuit S., Veuthey J.L., Zamboni N., Westermann B., Kunji E.R.S., Martinou J.C. (2012). Identification and functional expression of the mitochondrial pyruvate carrier. Science.

[36] Schell J.C., Rutter J. (2013). The long and winding road to the mitochondrial pyruvate carrier. Cancer Metab..

[37] Vanderperre B., Cermakova K., Escoffier J., Kaba M., Bender T., Nef S., Martinou J.C. (2016). MPC1-like Is a placental mammal-specific mitochondrial pyruvate carrier subunit expressed in postmeiotic male germ cells. J. Biol. Chem..

[38] Li C.L., Wang M., Ma X.Y., Zhang W. (2014). NRGA1, a putative mitochondrial pyruvate carrier, mediates ABA regulation of guard cell ion channels and drought stress responses in Arabidopsis. Mol. Plant.

[39] Shen J.L., Li C.L., Wang M., He L.L., Lin M.Y., Chen D.H., Zhang W. (2017). Mitochondrial pyruvate carrier 1 mediates abscisic acid-regulated stomatal closure and the drought response by affecting cellular pyruvate content in Arabidopsis thaliana. BMC Plant Biol..

[40] Orlandi I., Coppola D.P., Vai M. (2014). Rewiring yeast acetate metabolism through MPC1 loss of function leads to mitochondrial damage and decreases chronological lifespan. Microb. Cell.

[41] Vanderperre B., Herzig S., Krznar P., Hörl M., Ammar Z., Montessuit S., Pierredon S., Zamboni N., Martinou J.C. (2016). Embryonic lethality of mitochondrial pyruvate carrier 1 deficient mouse can be rescued by a ketogenic diet. PLoS Genet..

[42] Vigueira P.A., McCommis K.S., Schweitzer G.G., Remedi M.S., Chambers K.T., Fu X., McDonald W.G., Cole S.L., Colca J.R., Kletzien R.F. (2014). Mitochondrial pyruvate carrier 2 hypomorphism in mice leads to defects in glucose-stimulated insulin secretion. Cell Rep..

[43] Oonthonpan L., Rauckhorst A.J., Gray L.R., Boutron A.C., Taylor E.B. (2019). Two human patient mitochondrial pyruvate carrier mutations reveal distinct molecular mechanisms of dysfunction. JCI Insight.

[44] Nagampalli R.S.K., Quesñay J.E.N., Adamoski D., Islam Z., Birch J., Sebinelli H.G., Girard R.M.B.M., Ascenção C.F.R., Fala A.M., Pauletti B.A. (2018). Human mitochondrial pyruvate carrier 2 as an autonomous membrane transporter. Sci. Rep..

[45] Divakaruni A.S., Wiley S.E., Rogers G.W., Andreyev A.Y., Petrosyan S., Loviscach M., Wall E.A., Yadava N., Heuck A.P., Ferrick D.A. (2013). Thiazolidinediones are acute, specific inhibitors of the mitochondrial pyruvate carrier. Proc. Natl. Acad. Sci. USA.

[46] Tavoulari S., Thangaratnarajah C., Mavridou V., Harbour M.E., Martinou J.C., Kunji E.R. (2019). The yeast mitochondrial pyruvate carrier is a hetero-dimer in its functional state. EMBO J..

[47] Timón-Gómez A., Proft M., Pascual-Ahuir A. (2013). Differential regulation of mitochondrial pyruvate carrier genes modulates respiratory capacity and stress tolerance in yeast. PLoS ONE.

[48] Bender T., Pena G., Martinou J.C. (2015). Regulation of mitochondrial pyruvate uptake by alternative pyruvate carrier complexes. EMBO J..

[49] Patterson J.N., Cousteils K., Lou J.W., Manning Fox J.E., MacDonald P.E., Joseph J.W. (2014). Mitochondrial metabolism of pyruvate is essential for regulating glucose-stimulated insulin secretion. J. Biol. Chem..

[50] Lehmann J.M., Moore L.B., Smith-Oliver T.A., Wilkison W.O., Willson T.M., Kliewer S.A. (1995). An antidiabetic thiazolidinedione is a high affinity ligand for peroxisome proliferator-activated receptor gamma (PPAR gamma). J. Biol. Chem..

[51] Hauner H. (2002). The mode of action of thiazolidinediones. Diabetes Metab. Res. Rev..

[52] Colca J.R., McDonald W.G., Cavey G.S., Cole S.L., Holewa D.D., Brightwell-Conrad A.S., Wolfe C.L., Wheeler J.S., Coulter K.R., Kilkuskie P.M. (2013). Identification of a mitochondrial target of thiazolidinedione insulin sensitizers (mTOT)-relationship to newly identified mitochondrial pyruvate carrier proteins. PLoS ONE.

[53] Colca J.R., VanderLugt J.T., Adams W.J., Shashlo A., McDonald W.G., Liang J., Zhou R., Orloff D.G. (2013). Clinical proof-of-concept study with MSDC-0160, a prototype mTOT-modulating insulin sensitizer. Clin. Pharmacol. Ther..

[54] Chen Z., Vigueira P.A., Chambers K.T., Hall A.M., Mitra M.S., Qi N., McDonald W.G., Colca J.R., Kletzien R.F., Finck B.N. (2012). Insulin resistance and metabolic derangements in obese mice are ameliorated by a novel peroxisome proliferator-activated receptor γ-sparing thiazolidinedione. J. Biol. Chem..

[55] Warburg O., Wind F., Negelein E. (1927). The metabolism of tumors in the body. J. Gen. Physiol..

[56] Vander Heiden M.G., Cantley L.C., Thompson C.B. (2009). Understanding the Warburg effect: The metabolic requirements of cell proliferation. Science.

[57] Iqbal M.A., Gupta V., Gopinath P., Mazurek S., Bamezai R.N.K. (2014). Pyruvate kinase M2 and cancer: An updated assessment. FEBS Lett..

[58] Augoff K., Hryniewicz-Jankowska A., Tabola R. (2015). Lactate dehydrogenase 5: An old friend and a new hope in the war on cancer. Cancer Lett..

[59] Kim J.W., Tchernyshyov I., Semenza G.L., Dang C.V. (2006). HIF-1-mediated expression of pyruvate dehydrogenase kinase: A metabolic switch required for cellular adaptation to hypoxia. Cell Metab..

[60] Schell J.C., Olson K.A., Jiang L., Hawkins A.J., Van Vranken J.G., Xie J., Egnatchik R.A., Earl E.G., DeBerardinis R.J., Rutter J. (2014). A role for the mitochondrial pyruvate carrier as a repressor of the Warburg effect and colon cancer cell growth. Mol. Cell.

[61] Wang L., Xu M., Qin J., Lin S.C., Lee H.J., Tsai S.Y., Tsai M.J. (2016). MPC1, a key gene in cancer metabolism, is regulated by COUPTFII in human prostate cancer. Oncotarget.

[62] Li X., Ji Y., Han G., Li X., Fan Z., Li Y., Zhong Y., Cao J., Zhao J., Zhang M. (2016). MPC1 and MPC2 expressions are associated with favorable clinical outcomes in prostate cancer. BMC Cancer.

[63] Li Y., Li X., Kan Q., Zhang M., Li X., Xu R., Wang J., Yu D., Goscinski M.A., Wen J.G. (2017). Mitochondrial pyruvate carrier function is negatively linked to Warburg phenotype in vitro and malignant features in esophageal squamous cell carcinomas. Oncotarget.

[64] Schell J.C., Wisidagama D.R., Bensard C., Zhao H., Wei P., Tanner J., Flores A., Mohlman J., Sorensen L.K., Earl C.S. (2017). Control of intestinal stem cell function and proliferation by mitochondrial pyruvate metabolism. Nat. Cell Biol..

[65] Zou H., Chen Q., Zhang A., Wang S., Wu H., Yuan Y., Wang S., Yu J., Luo M., Wen X. (2019). MPC1 deficiency accelerates lung adenocarcinoma progression through the STAT3 pathway. Cell Death Dis..

[66] Takaoka Y., Konno M., Koseki J., Colvin H., Asai A., Tamari K., Satoh T., Mori M., Doki Y., Ogawa K. (2019). Mitochondrial pyruvate carrier 1 expression controls cancer epithelial-mesenchymal transition and radioresistance. Cancer Sci..

[67] Bader D.A., Hartig S.M., Putluri V., Foley C., Hamilton M.P., Smith E.A., Saha P.K., Panigrahi A., Walker C., Zong L. (2019). Mitochondrial pyruvate import is a metabolic vulnerability in androgen receptor-driven prostate cancer. Nat. Metab..

[68] Tang X.P., Chen Q., Li Y., Wang Y., Zou H.B., Fu W.J., Niu Q., Pan Q.G., Jiang P., Xu X.S. (2019). Mitochondrial pyruvate carrier 1 functions as a tumor suppressor and predicts the prognosis of human renal cell carcinoma. Lab. Investig..

[69] Zhong Y., Li X., Yu D., Li X., Li Y., Long Y., Yuan Y., Ji Z., Zhang M., Wen J.G. (2015). Application of mitochondrial pyruvate carrier blocker UK5099 creates metabolic reprogram and greater stem-like properties in LnCap prostate cancer cells in vitro. Oncotarget.

[70] Li X., Han G., Li X., Kan Q., Fan Z., Li Y., Ji Y., Zhao J., Zhang M., Grigalavicius M. (2017). Mitochondrial pyruvate carrier function determines cell stemness and metabolic reprogramming in cancer cells. Oncotarget.

[71] Ohashi T., Eguchi H., Kawamoto K., Konno M., Asai A., Colvin H., Ueda Y., Takaoka H., Iwagami Y., Yamada D. (2018). Mitochondrial pyruvate carrier modulates the epithelial-mesenchymal transition in cholangiocarcinoma. Oncol. Rep..

[72] Corbet C., Bastien E., Draoui N., Doix B., Mignion L., Jordan B.F., Marchand A., Vanherck J.C., Chaltin P., Schakman O. (2018). Interruption of lactate uptake by inhibiting mitochondrial pyruvate transport unravels direct antitumor and radiosensitizing effects. Nat. Commun..

[73] Owen O.E., Kalhan S.C., Hanson R.W. (2002). The key role of anaplerosis and cataplerosis for citric acid cycle function. J. Biol. Chem..

[74] Yang C., Sudderth J., Dang T., Bachoo R.M., Bachoo R.G., McDonald J.G., DeBerardinis R.J. (2009). Glioblastoma cells require glutamate dehydrogenase to survive impairments of glucose metabolism or Akt signaling. Cancer Res..

[75] Yang C., Ko B., Hensley C.T., Jiang L., Wasti A.T., Kim J., Sudderth J., Calvaruso M.A., Lumata L., Mitsche M. (2014). Glutamine oxidation maintains the TCA cycle and cell survival during impaired mitochondrial pyruvate transport. Mol. Cell.

[76] Vacanti N.M., Divakaruni A.S., Green C.R., Parker S.J., Henry R.R., Ciaraldi T.P., Murphy A.N., Metallo C.M. (2014). Regulation of substrate utilization by the mitochondrial pyruvate carrier. Mol. Cell.

[77] Gray L.R., Sultana M.R., Rauckhorst A.J., Oonthonpan L., Tompkins S.C., Sharma A., Fu X., Miao R., Pewa A.D., Brown K.S. (2015). Hepatic mitochondrial pyruvate carrier 1 is required for efficient regulation of gluconeogenesis and whole-body glucose homeostasis. Cell Metab..

[78] McCommis K.S., Chen Z., Fu X., McDonald W.G., Colca J.R., Kletzien R.F., Burgess S.C., Finck B.N. (2015). Loss of mitochondrial pyruvate carrier 2 in the liver leads to defects in gluconeogenesis and compensation via pyruvate-alanine cycling. Cell Metab..

[79] Sharma A., Oonthonpan L., Sheldon R.D., Rauckhorst A.J., Zhu Z., Tompkins S.C., Cho K., Grzesik W.J., Gray L.R., Scerbo D.A. (2019). Impaired skeletal muscle mitochondrial pyruvate uptake rewires glucose metabolism to drive whole-body leanness. eLife.

[80] Nordströma U., Beauvais G., Ghosh A., Pulikkaparambil Sasidharan B.C., Lundblad M., Fuchs J., Joshi R.L., Lipton J.W., Roholt A., Medicetty S. (2015). Progressive nigrostriatal terminal dysfunction and degeneration in the engrailed1 heterozygous mouse model of Parkinson’s disease. Neurobiol. Dis..

[81] Perluigi M., Di Domenico F., Butterfield D.A. (2015). mTOR signaling in aging and neurodegeneration: At the crossroad between metabolism dysfunction and impairment of autophagy. Neurobiol. Dis..

[82] Jahrling J.B., Laberge R.M. (2015). Age-related neurodegeneration prevention through mTOR inhibition: Potential mechanisms and remaining questions. Curr. Top. Med. Chem..

[83] Frake R.A., Ricketts T., Menzies F.M., Rubinsztein D.C. (2015). Autophagy and neurodegeneration. J. Clin. Investig..

[84] Dello Russo C., Lisi L., Tringali G., Navarra P. (2009). Involvement of mTOR kinase in cytokine-dependent microglial activation and cell proliferation. Biochem. Pharmacol..

[85] Li D., Wang C., Yao Y., Chen L., Liu G., Zhang R., Liu Q., Shi F.D., Hao J. (2016). mTORC1 pathway disruption ameliorates brain inflammation following stroke via a shift in microglia phenotype from M1 type to M2 type. FASEB J..

[86] Srivastava I.N., Shperdheja J., Baybis M., Ferguson T., Crino P.B. (2016). mTOR pathway inhibition prevents neuroinflammation and neuronal death in a mouse model of cerebral palsy. Neurobiol. Dis..

[87] McKenna M.C., Stridh M.H., McNair L.F., Sonnewald U., Waagepetersen H.S., Schousboe A. (2016). Glutamate oxidation in astrocytes: Roles of glutamate dehydrogenase and aminotransferases. J. Neurosci. Res..

[88] Hertz L., Rothman D.L. (2017). Glutamine-glutamate cycle flux is similar in cultured astrocytes and brain and both glutamate production and oxidation are mainly catalyzed by Aspartate Aminotransferase. Biology.

[89] Du J., Cleghorn W.M., Contreras L., Lindsay K., Rountree A.M., Chertov A.O., Turner S.J., Sahaboglu A., Linton J., Sadilek M. (2013). Inhibition of mitochondrial pyruvate transport by zaprinast causes massive accumulation of aspartate at the expense of glutamate in the retina. J. Biol. Chem..

[90] Saxton R.A., Sabatini D.M. (2017). mTOR signaling in growth, metabolism, and disease. Cell.

[91] Hardie D.G. (2014). AMPK: Positive and negative regulation, and its role in whole-body energy homeostasis. Curr. Opin. Cell Biol..

[92] Gwinn D.M., Shackelford D.B., Egan D.F., Mihaylova M.M., Mery A., Vasquez D.S., Turk B.E., Shaw R.J. (2008). AMPK phosphorylation of raptor mediates a metabolic checkpoint. Mol. Cell.

[93] Inoki K., Zhu T., Guan K.L. (2003). TSC2 mediates cellular energy response to control cell growth and survival. Cell.

[94] Han J.M., Sahin M. (2011). TSC1/TSC2 signaling in the CNS. FEBS Lett..

[95] Zhou G., Myers R., Li Y., Chen Y., Shen X., Fenyk-Melody J., Wu M., Ventre J., Doebber T., Fujii N. (2001). Role of AMP-activated protein kinase in mechanism of metformin action. J. Clin. Investig..

[96] Marinangeli C., Didier S., Vingtdeux V. (2016). AMPK in neurodegenerative diseases: Implications and therapeutic perspectives. Curr. Drug Targets.

[97] Rotermund C., Machetanz G., Fitzgerald J.C. (2018). The therapeutic potential of Metformin in neurodegenerative diseases. Front. Endocrinol..

[98] Gantois I., Popic J., Khoutorsky A., Sonenberg N. (2019). Metformin for treatment of Fragile X Syndrome and other neurological disorders. Annu. Rev. Med..

[99] Felig P. (1973). The glucose-alanine cycle. Metabolism.

[100] Corbet C., Feron O. (2015). Metabolic and mind shifts: From glucose to glutamine and acetate addictions in cancer. Curr. Opin. Clin. Nutr. Metab. Care.

[101] Goubert E., Mircheva Y., Lasorsa F.M., Melon C., Profilo E., Sutera J., Becq H., Palmieri F., Palmieri L., Aniksztejn L. (2017). Inhibition of the mitochondrial glutamate carrier SLC25A22 in astrocytes leads to intracellular glutamate accumulation. Front. Cell. Neurosci..

[102] Mandrekar-Colucci S., Karlo J.C., Landreth G.E. (2012). Mechanisms underlying the rapid peroxisome proliferator-activated receptor-γ-mediated amyloid clearance and reversal of cognitive deficits in a murine model of Alzheimer’s disease. J. Neurosci..

[103] Pérez M.J., Quintanilla R.A. (2015). Therapeutic actions of the Thiazolidinediones in Alzheimer’s disease. PPAR Res..

[104] Chen J., Li S., Sun W., Li J. (2015). Anti-diabetes drug pioglitazone ameliorates synaptic defects in AD transgenic mice by inhibiting cyclin-dependent kinase5 activity. PLoS ONE.

[105] Galimberti D., Scarpini E. (2017). Pioglitazone for the treatment of Alzheimer’s disease. Expert Opin. Investig. Drugs.

[106] Brauer R., Bhaskaran K., Chaturvedi N., Dexter D.T., Smeeth L., Douglas I. (2015). Glitazone treatment and incidence of Parkinson’s disease among people with diabetes: A retrospective cohort study. PLoS Med..

[107] NINDS Exploratory Trials in Parkinson Disease (NET-PD) FS-ZONE Investigators (2015). Pioglitazone in early Parkinson’s disease: A phase 2, multicentre, double-blind, randomised trial. Lancet Neurol..

[108] Connolly J.G., Bykov K., Gagne J.J. (2015). Thiazolidinediones and Parkinson disease: A cohort study. Am. J. Epidemiol..

[109] Pinto M., Nissanka N., Peralta S., Brambilla R., Diaz F., Moraes C.T. (2016). Pioglitazone ameliorates the phenotype of a novel Parkinson’s disease mouse model by reducing neuroinflammation. Mol. Neurodegener..

[110] Brakedal B., Flønes I., Reiter S.F., Torkildsen Ø., Dölle C., Assmus J., Haugarvoll K., Tzoulis C. (2017). Glitazone use associated with reduced risk of Parkinson’s disease. Mov. Disord..

[111] Schütz B., Reimann J., Dumitrescu-Ozimek L., Kappes-Horn K., Landreth G.E., Schürmann B., Zimmer A., Heneka M.T. (2005). The oral antidiabetic pioglitazone protects from neurodegeneration and amyotrophic lateral sclerosis-like symptoms in superoxide dismutase-G93A transgenic mice. J. Neurosci..

[112] Joardar A., Menzl J., Podolsky T.C., Manzo E., Estes P.S., Ashford S., Zarnescu D.C. (2015). PPAR gamma activation is neuroprotective in a Drosophila model of ALS based on TDP-43. Hum. Mol. Genet..

[113] Chiang M.C., Chern Y., Huang R.N. (2012). PPARgamma rescue of the mitochondrial dysfunction in Huntington’s disease. Neurobiol. Dis..

[114] Jin J., Albertz J., Guo Z., Peng Q., Rudow G., Troncoso J.C., Ross C.A., Duan W. (2013). Neuroprotective effects of PPAR-γ agonist rosiglitazone in N171-82Q mouse model of Huntington’s disease. J. Neurochem..

[115] Chiang M.C., Cheng Y.C., Nicol C.J., Lin K.H., Yen C.H., Chen S.J., Huang R.N. (2015). Rosiglitazone activation of PPARγ-dependent signaling is neuroprotective in mutant huntingtin expressing cells. Exp. Cell Res..

[116] Leszek J., Trypka E., Tarasov V.V., Ashraf G.M., Aliev G. (2017). Type 3 Diabetes Mellitus: A novel implication of Alzheimers disease. Curr. Top. Med. Chem..

[117] Pearson-Leary J., McNay E.C. (2016). Novel roles for the insulin-regulated Glucose Transporter-4 in hippocampally dependent memory. J. Neurosci..

[118] Spinelli M., Fusco S., Grassi C. (2019). Brain insulin resistance and hippocampal plasticity: Mechanisms and biomarkers of cognitive decline. Front. Neurosci..

[119] Craig A., Parvez F., Issberner J. (2019). A systematic literature review of the effect of insulin sensitizers on the cognitive symptoms of Alzheimer’s Disease in transgenic mice. Behav. Brain Res..

[120] Morgan C.L., Inzucchi S.E., Puelles J., Jenkins-Jones S., Currie C.J. (2018). Impact of treatment with pioglitazone on stroke outcomes: A real-world database analysis. Diabetes Obes. Metab..

[121] Lee M., Saver J.L., Liao H.W., Lin C.H., Ovbiagele B. (2017). Pioglitazone for secondary stroke prevention: A systematic review and meta-analysis. Stroke.

[122] Yaghi S., Furie K.L., Viscoli C.M., Kamel H., Gorman M., Dearborn J., Young L.H., Inzucchi S.E., Lovejoy A.M., Kasner S.E. (2018). Pioglitazone prevents stroke in patients with a recent transient ischemic attack or ischemic stroke: A planned secondary analysis of the IRIS trial (Insulin Resistance Intervention After Stroke). Circulation.

[123] McGuire J.L., Correll E.A., Lowery A.C., Rhame K., Anwar F.N., McCullumsmith R.E., Ngwenya L.B. (2019). Pioglitazone improves working memory performance when administered in chronic TBI. Neurobiol. Dis..

[124] Phelix C., Bourdon A., Dugan J., Villareal G., Perry G. (2017). MSDC-0160 and MSDC-0602 binding with human mitochondrial pyruvate carrier (MPC) 1 and 2 heterodimer: PPARγ activating and sparing TZDs as therapeutics. Int. J. Knowl. Knowl. Bioinform..

[125] Nancolas B., Guo L., Zhou R., Nath K., Nelson D.S., Leeper D.B., Blair I.A., Glickson J.D., Halestrap A.P. (2016). The anti-tumour agent lonidamine is a potent inhibitor of the mitochondrial pyruvate carrier and plasma membrane monocarboxylate transporters. Biochem. J..

[126] Mansi J.L., de Graeff A., Newell D.R., Glaholm J., Button D., Leach M.O., Payne G., Smith I.E. (1991). A phase II clinical and pharmacokinetic study of Lonidamine in patients with advanced breast cancer. Br. J. Cancer.

[127] Cheng G., Zhang Q., Pan J., Lee Y., Ouari O., Hardy M., Zielonka M., Myers C.R., Zielonka J., Weh K. (2019). Targeting lonidamine to mitochondria mitigates lung tumorigenesis and brain metastasis. Nat. Commun..

[128] Di Cosimo S., Ferretti G., Papaldo P., Carlini P., Fabi A., Cognetti F. (2003). Lonidamine: Efficacy and safety in clinical trials for the treatment of solid tumors. Drugs Today.

